# Bedside Assessment of the Respiratory System During Invasive Mechanical Ventilation

**DOI:** 10.3390/jcm13237456

**Published:** 2024-12-07

**Authors:** Lorenzo Giosa, Patrick D. Collins, Sridevi Shetty, Marta Lubian, Riccardo Del Signore, Mara Chioccola, Francesca Pugliese, Luigi Camporota

**Affiliations:** 1 Department of Critical Care Medicine, Guy’s and St Thomas’ NHS Foundation Trust, London SE1 7EH, UK; 2 Center for Human and Applied Physiological Sciences, School of Basic and Medical Biosciences, King’s College London, London WC2R 2LS, UK

**Keywords:** mechanical ventilation, respiratory physiology, acute respiratory distress syndrome

## Abstract

Assessing the respiratory system of a patient receiving mechanical ventilation is complex. We provide an overview of an approach at the bedside underpinned by physiology. We discuss the importance of distinguishing between extensive and intensive ventilatory variables. We outline methods to evaluate both passive patients and those making spontaneous respiratory efforts during assisted ventilation. We believe a comprehensive assessment can influence setting mechanical ventilatory support to achieve lung and diaphragm protective ventilation.

## 1. Introduction

During invasive mechanical ventilation, a complex interaction takes place between the energy delivered by the ventilator, the contraction of the respiratory muscles, and the resulting stress and strain in the lung tissue. The primary goal for clinicians is to minimise ventilation-induced lung injury (VILI), while ideally maintaining sufficient spontaneous respiratory effort to preserve muscle strength and diaphragm function.

In the absence of strong evidence-based guidelines, clinicians currently rely on secondary evidence (e.g., *post hoc* analyses of clinical trials [1,2] or physiological studies [3]) to set the ventilator according to specific variables and proposed safety threshold: e.g., driving pressure (∆P) < 15 cm H_2_O [1] or mechanical power (MP) < 17 J/min [2]. While this approach may appear straightforward, measuring ventilatory variables and interpreting waveforms can be challenging in clinical practice. Additionally, there are significant differences between setting ventilation in fully sedated patients under mandatory modes and those breathing spontaneously on assisted modes.

This review offers practical guidance on assessing the respiratory system at the bedside under both types of mechanical ventilation, focusing on the accurate measurement and interpretation of key ventilatory variables to prevent lung injury and ensure adequate respiratory effort. Specifically, during invasive mechanical ventilation, excessive pressure and volume changes over time can cause ventilator-induced lung injury (VILI), while strong spontaneous efforts may lead to patient self-inflicted lung injury (P-SILI). The purpose of this manuscript is to guide the assessment of VILI and P-SILI risk to enable lung and diaphragm protective ventilation. Of note, protective ventilatory strategies must be balanced with gas-exchange optimisation. Namely, oxygenation targets, permissive hypercapnia, and assessment of ventilation–perfusion relationships represent crucial challenges during mechanical ventilation, which will not be discussed here. For comprehensive discussion of those subjects, we refer readers to excellent existing reviews [4,5,6].

### Ventilator Variables: Extensive vs. Intensive

Ventilatory variables can be schematically divided into *extensive* and *intensive* depending on their relationship to lung size, i.e., the functional residual capacity (FRC). This distinction is important, as it affects the significance of ventilatory variables and their relationship to VILI.

If we assume that a lung, whether healthy or diseased, is made up of alveoli with a fixed volume (approximately 4.2 × 10^−6^ mL), then the size of the lung (the FRC) correlates closely with the number of alveoli [7]. *Extensive* ventilatory variables are those whose effect on alveolar stretch, and therefore potential for VILI, depends on the total number of alveoli. An example is tidal volume ($V_{t}$): the same volume applied to a smaller lung (i.e., a lung with fewer alveoli) produces more stretch for each alveolus, and therefore has a different impact on VILI in healthy vs. diseased “baby lungs” [8]. Conversely, *intensive* ventilatory variables affect alveolar stretch independently of the number of alveoli. An example is driving pressure (∆P): outside severe lung inhomogeneity [9] whatever the number of alveoli, each alveolar unit theoretically receives the same amount of pressure, and therefore undergoes similar deformation. This is because ∆P “normalises” $V_{t}$ to the size of the lung through the static compliance of the respiratory system ($C_{stat}$) [1], which correlates closely with the FRC [10,11]:

(1) $$∆P=\frac{V_{t}}{C_{stat}}$$

Throughout this manuscript, we will differentiate between extensive and intensive ventilatory variables (Table 1) to guide their interpretation and relationship to lung injury.

## 2. Mandatory Ventilation in Passive Patients

In the early stages of respiratory failure, patients are typically fully sedated, and often paralysed, requiring ventilation in mandatory modes. In this state, there is no spontaneous respiratory effort, meaning the ventilatory settings entirely determine the risk of VILI. Key factors to consider include driving pressure (ΔP), positive end-expiratory pressure (PEEP), mechanical power (MP), and transpulmonary pressures. This section outlines how these ventilatory variables can be measured and interpreted in clinical practice.

### 2.1. Assessing the Inspiratory Phase

**Figure 1:**
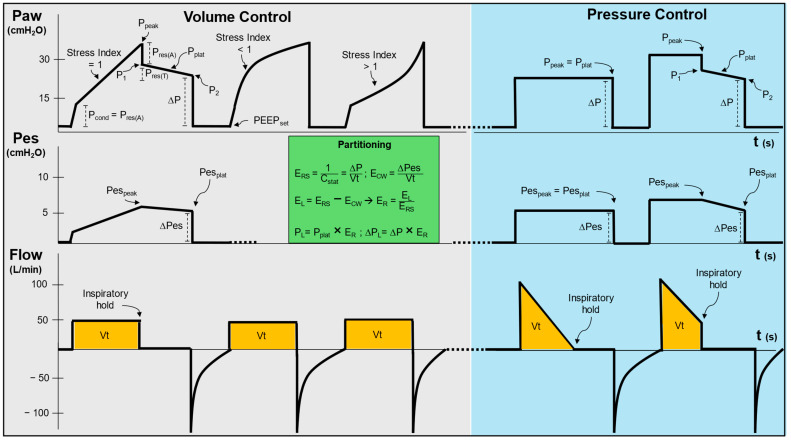
Assessment of the inspiratory phase. Volume control with constant (square) flow: the first breath describes the static conditions of an inspiratory hold required to assess driving and resistive pressures. Notably, the conductive pressure equals the airway resistive pressure, indicating the absence of airway closure; the second and third breaths describe dynamic conditions with negative and positive stress indexes, respectively. Pressure control with decelerating flow: both breaths describe the static conditions of an inspiratory hold. However, in the first one the flow reaches zero before the hold, therefore peak and plateau pressures are synonymous, while a resistive decay is appreciated in the second breath as the flow is still positive when the hold is performed. The green “Partitioning” box describes how to convert airway into transpulmonary pressures with the “elastance derived” method using oesophageal pressures. P_aw_: airway pressure, P_peak_: peak inspiratory pressure, P_res(A)_: airway resistive pressure, P_res(T)_: tissue resistive pressure, ∆P: driving pressure, P_plat_: plateau pressure, P_cond_: conductive pressure, PEEP_set_: set positive end-expiratory pressure, Pes: oesophageal pressure, Pes_peak_: peak inspiratory oesophageal pressure, Pes_plat_: plateau oeasophageal pressure, ∆Pes: driving oesophageal pressure, E_rs_: respiratory system elastance, C_stat_: static respiratory system compliance; Vt: tidal volume; E_cw_: chest wall elastance, E_L_: lung elastance, E_R_: elastance ratio, P_L_: inspiratory transpulmonary pressure, ∆P_L_: driving transpulmonary pressure.

Figure 1 illustrates the assessment of the inspiratory phase during volume or pressure control modes, which use constant or decelerating flow patterns, respectively.

#### 2.1.1. Static Conditions

Performing an inspiratory occlusion manoeuvre allows the assessment of inspiration under static conditions: in volume control ventilation, airway pressure falls from its peak (P_peak_) to a first nadir (P_1_) before reaching a plateau (P_2_). The difference between P_peak_ and P_1_ is the *airway* resistive pressure (P_res(A)_), while the difference between P_1_ and P_2_ represents the *tissue* resistive pressure (P_res(T)_). The latter usually reflects a viscoelastic property of the respiratory system named *stress relaxation* [12] and, in the absence of instrumental leaks, indicates lung inhomogeneity [13]. As P_1_ and P_2_ are often similar in clinical practice, the pressure value taken ~0.5 s from the inspiratory hold is generally considered reflective of the plateau pressure (P_plat_) [14]. Conversely, in pressure control mode with decelerating flow, P_peak_ and P_plat_ are generally the same provided the inspiratory flow has reached *zero* at the end of the inspiratory time [15] (Figure 1). In both modes, ∆P is calculated as the difference between P_plat_ and the total positive end-expiratory pressure (PEEP), calculated at the end of an end-expiratory hold manoeuvre (see below) [16]. As detailed above, learning how to accurately calculate this variable is important, as ∆P represents the *intensive* variable describing how much the achieved Vt (an *extensive* variable) “stretches” the respiratory system above its resting volume (i.e., the FRC) [1].

#### 2.1.2. Dynamic Conditions

In volume-controlled ventilation (with square wave, or constant flow), additional information can be obtained in dynamic conditions, i.e., outside an inspiratory hold: *first*, the difference between PEEP and the pressure at which an initial inspiratory slope change in the pressure waveform occurs is referred to as conductive pressure (P_cond_) and normally reflects the pressure necessary to overcome airway resistance. Accordingly, P_cond_ should equal P_res(A)_ (Figure 1), unless airway closure is present (see below) [17]; *second*, the slope of the pressure waveform beyond P_cond_ is indicative of two possible phenomena: (1) tidal recruitment (inspiratory opening of previously closed alveoli), or (2) dynamic overinflation. This behaviour can be described mathematically as the “stress index”:

(2) $$P_{aw}=a+t^{b}+c$$

where a is a scaling coefficient, c is the baseline pressure, and b is the stress index (the exponent of time t) which characterises the shape of the pressure–time curve.

However, this can also be assessed visually (Figure 1). A constant linear pressure slope suggests an overall balance between the two phenomena (or their absence) across the whole ventilated lung units. In this case the pressure–time relationship (pressure = ƒ (time)) is linear, i.e., the exponent of the variable *time* (normally referred to as stress index [18]) equals 1. Conversely, a non-linear slope suggests a prevalence of tidal recruitment if the exponent is <1 (upward convexity) or dynamic overinflation if >1 (upward concavity) (Figure 1). These situations might be associated with VILI in the form of atelecto- or volutrauma, respectively. Accordingly, the aim of this method is to set PEEP and Vt to achieve a stress index ≈ 1 [19].

### 2.2. Assessing the Expiratory Phase

**Figure 2:**
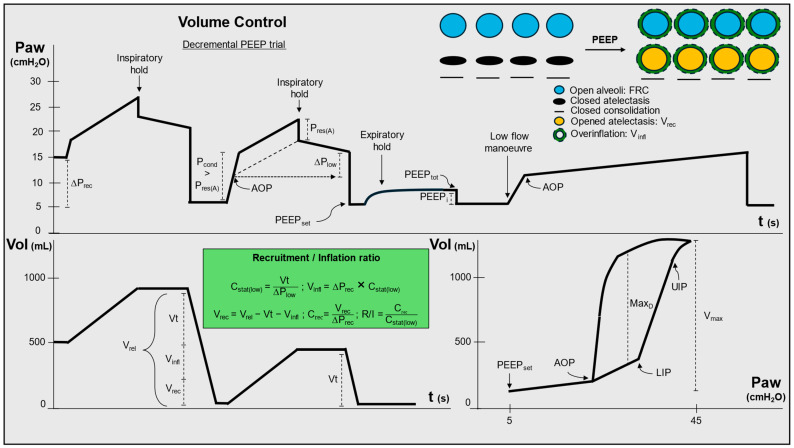
Assessment of the expiratory phase. The first two breaths describe a decremental PEEP trial (from 15 to 5 cm H_2_O) to calculate the recruitment to inflation ratio in volume control ventilation with constant (square) flow. Note that the second breath shows the presence of airway opening pressure and of intrinsic PEEP, the latter visualised during an expiratory hold. The driving pressure is calculated from the airway opening pressure, being higher than the set and total PEEP. Airway opening pressure can be assessed with three methods: (1) AOP is detected when P_cond_ is significantly higher than P_res_. The AOP value is defined as: AOP = PEEP + (P_cond_ − P_res_); (2) as the change in slope in a low-flow (<10 L/min) pressure–time curve (third breath); (3) as the beginning of inflation during the corresponding low-flow pressure–volume curve. P_aw_: airway pressure, Vol: volume, ∆P_rec_: change in PEEP during a decremental PEEP trial; P_res(A)_: airway resistive pressure, P_cond_: conductive pressure, AOP: airway opening pressure, ∆P_low_: driving pressure at the lower PEEP, PEEP_set_: set positive end-expiratory pressure, PEEP_i_: intrinsic positive end-expiratory pressure, PEEP_tot_: total positive end-expiratory pressure, V_rel_: release volume during a decremental PEEP trial, Vt: tidal volume, V_infl_: PEEP-induced inflation volume, V_rec_: PEEP-induced recruited volume; FRC: functional residual capacity, C_stat(low)_: static respiratory system compliance at the lower PEEP, C_rec_: compliance of the recruited volume, R/I: recruitment to inflation ratio, LIP: lower inflection point, UIP: upper inflection point; Max_D_: maximal distance between inspiratory and expiratory limb of a low-flow pressure–volume loop, V_max_: maximum volume inflated during a low-flow manoeuvre.

Figure 2 illustrates the assessment of the expiratory phase.

#### 2.2.1. Airway Closure (Gas Trapping)

Ideally, the assessment of the expiratory phase should begin by checking for the presence of an airway opening pressure (AOP). Complete closure of the airways—reversible with sufficient positive pressure—has been found in ~20–25% of obese patients undergoing general anaesthesia [20] and 25–33% of patients with ARDS [21] (perhaps the majority with obesity [22]) or pulmonary oedema [23]. The AOP is simply the pressure required to open the lower airways of the patient which have been collapsed by the weight of the chest wall and lung tissue. At pressures below the AOP (if present), there is no communication between the endotracheal tube and the alveoli of the patient, and AOP should be viewed as a “lower limit” when setting PEEP, as any PEEP below the AOP will not be transmitted to the alveoli [21]. Different possibilities exist to assess for the presence of AOP, either the P_cond_ being higher than the P_res(A)_ in volume control mode with constant flow or the evidence of a sharp change in compliance during a low-flow pressure–volume (PV) loop manoeuvre where the first compliance slope below the AOP is the compliance of the tubing and above the AOP the slope is the compliance of the respiratory system [17,21,23,24] (Figure 2). This is most intuitive using a low-flow PV loop: below the AOP, increases in pressure result in minimal changes in volume. This reflects only distension of the ventilator apparatus and artificial airway (which have a compliance of around 2.5 mL/cm H_2_O). When the AOP is reached, a step change occurs as pressure is now transmitted past the opened lower airways and can distend the respiratory system.

#### 2.2.2. Intrinsic PEEP

A second step is represented by the evaluation of intrinsic PEEP (PEEP_i_). This occurs when there is incomplete time for expiration between breaths, due to expiratory flow limitations imposed by airway pathology, circuit obstruction, or inappropriate ventilation settings [25]. In passive patients, PEEP_i_ can be assessed with an end-expiratory hold. Recognition of PEEP_i_ (the expiratory pressure that adds to PEEP_set_) may have implications for setting the ventilator [26]: if a flow limitation is present due to collapsed airways (e.g., in chronic obstructive pulmonary disease), PEEP_set_ may help prevent their closure when set at ~80% of the PEEP_tot_ [27]. Conversely, when the flow limitation is due to increased expiratory resistance rather than airway collapse (e.g., in acute asthma), low PEEP, ≤5 cm H_2_O, is normally used or the PEEP that does not lead to an increase in plateau pressure [28,29]. More importantly, detection of AOP and PEEP_i_ is also essential for the correct calculation of ∆P, as the latter should be computed as the difference between P_plat_ and the highest value among PEEP_set_, PEEP_tot_, and AOP (Figure 2).

#### 2.2.3. Recruitment and Overdistension

Once assessed for AOP and PEEP_i_, the effects of PEEP on recruitment/overdistension should be investigated. Of note, recruitment is mostly an inspiratory process. In other words, the pressure required to “open” previously closed alveoli is generally reached during inflation. However, since the closing pressure of recruitable alveoli is often lower than their opening pressure [30], PEEP can prevent expiratory de-recruitment and therefore “atelectotrauma”—repetitive cyclic opening and closing of alveoli [31]. A concomitant overdistension of open alveoli may occur, and the balance between the two phenomena (recruitment and overdistension) is what should guide setting of inspiratory pressure and PEEP at the bedside. In other words, PEEP is an *intensive* variable associated with a volume of aeration (*extensive* variable) which can be divided into two separate components: a recruited volume (V_rec_) due to opening of previously closed alveoli that do not undergo expiratory closure and a PEEP-induced inflation volume (V_infl_) due to increased inflation of open alveoli [32] (Figure 2). While V_infl_ increases inspiratory strain, i.e., adds to Vt “stretching” the lung above FRC, increasing the risk of VILI, V_rec_ increases FRC and decreases atelectotrauma by limiting the expiratory collapse of recruitable alveoli [33,34].

(3) $$Strain=\frac{Vt+V_{infl}\;\;}{FRC+V_{rec}}$$

Although recruitment can be visualised and quantified using computed tomography (CT) performed at different PEEP levels, distinguishing V_rec_ from V_infl_ is more challenging at the bedside, and several methods have been proposed:

1. *The best compliance method*

When PEEP is applied, the end-expiratory lung volume is represented by FRC + V_rec_ and can be approximated by the static compliance (C_stat_) measured at a given PEEP. Consequently, the inflation volume V_infl_ following an increase in PEEP can be estimated as ∆PEEP multiplied by C_stat_:

(4) $$V_{infl}=∆PEEP\cdot C_{stat}=∆PEEP\cdot \frac{Vt}{∆P}$$

From this equation, one can appreciate that (1) the higher the PEEP, the higher the V_infl_ and, therefore, the *static* stretch of the lung; however, (2) the higher the V_rec_ associated with the change in PEEP, the higher the end-expiratory lung volume and the lower the *dynamic* stretch (i.e., the ∆P required to obtain the desired Vt) [34]. This represents the reasoning behind setting PEEP with the “best compliance” method [35,36], whereby, during a decremental PEEP trial, the PEEP associated with the highest C_stat_ is suggested to reflect a better increase in V_rec_ than V_infl_.

However, a major limitation is that, since C_stat_ is measured during *inspiration*, with the best compliance method one cannot distinguish between improvements in C_stat_ due to sustained recruitment (beneficial), avoidance of overdistension (beneficial), or increased atelectotrauma (harmful) [37]. Indeed, during a decremental PEEP trial, if the patient has a high degree of recruitability, lower PEEP may paradoxically improve compliance by facilitating expiratory collapse of alveoli (atelectotrauma). This is because the proportional change in volume when alveoli snap open then completely close is larger than when an already open alveoli are distended more [30,38,39]. This may in part account for the worse ARDS outcomes seen in patients who received PEEP set according to the best compliance method during a decremental PEEP manoeuvre [40]. Notably, as stated above, the “tidal recruitment” associated with atelectotrauma might be suspected with stress index <1.

2. *The recruitment to inflation ratio*

Although the increase in compliance seen when alveoli snap open is a limitation in setting PEEP according to the best compliance method, it is a useful phenomenon to estimate the recruitability of a patient using the recruitment-to-inflation ratio (R/I). This method uses a single-breath manoeuvre from higher to lower PEEP to assess the effects of PEEP on V_rec_ and V_infl_ [24]. The first exhaled breath after the PEEP change includes both the set Vt and the volume trapped by the ∆PEEP. As the V_rec_ has a higher compliance than Vi_nfl_, the R/I ratio divides the compliance of the V_rec_ by the C_stat_ at low PEEP. A higher ratio indicates a greater V_rec_ induced by PEEP. The manoeuvre is described in Figure 2, and an online calculator is available for bedside application (https://respiratorycalc.com/ri-ratio accessed on 28 November 2024). An R/I of <0.5 (meaning that most of the volume gained with PEEP reflects overdistension of open alveoli rather than recruitment) suggests that the use of higher PEEP is unlikely to be beneficial.

3. *Low-flow PV loops*

Inspection of low-flow pressure–volume (PV) loops is also informative of the effects of PEEP on lung mechanics. Indeed, aside from AOP, PV loops allow for detection of the lower and upper inflection points (LIP and UIP), classically viewed as the pressure marking the end of recruitment and the beginning of overinflation, respectively [41] (Figure 2). However, it is likely that there is a range of recruited or overinflated alveoli across much of the inspiratory limb, therefore the LIP may simply mark the beginning of *significant* recruitment and the UIP the end of *significant* recruitment. As PEEP is an expiratory phenomenon, it may be best set accounting for information from the expiratory phase. Accordingly, during a low-flow PV loop, the hysteresis area (the area between the inspiratory and expiratory P-V limbs) correlates with the degree of recruitability on CT. For example, where the largest normalised volume difference (Max_D_) between the two limbs is >41% of the inspiratory capacity reached at 40 cmH_2_O (V_max_), there is likely to be significant recruitability and potential benefit from a higher PEEP strategy [42] (Figure 2).

It is noteworthy that both the recruitment-to-inflation ratio and the hysteresis of a 0–40 cmH_2_O low-flow PV loop give information regarding the extent of recruitability (i.e., the V_rec_ associated with the highest tested pressure, an extensive property) but not the specific “optimal” PEEP value exceeding the closing pressure of recruitable lung units (an intensive property) [43]. How to best assess the latter at the bedside remains an open question [34]. However, applying high PEEP to patients with low recruitability may be harmful [44]. Indeed, just as patients with low recruitability tend to experience overdistension with higher PEEP, they also suffer greater cardiovascular adverse effects from high PEEP [45].

### 2.3. Assessing the Entire Breath over Time

**Figure 3:**
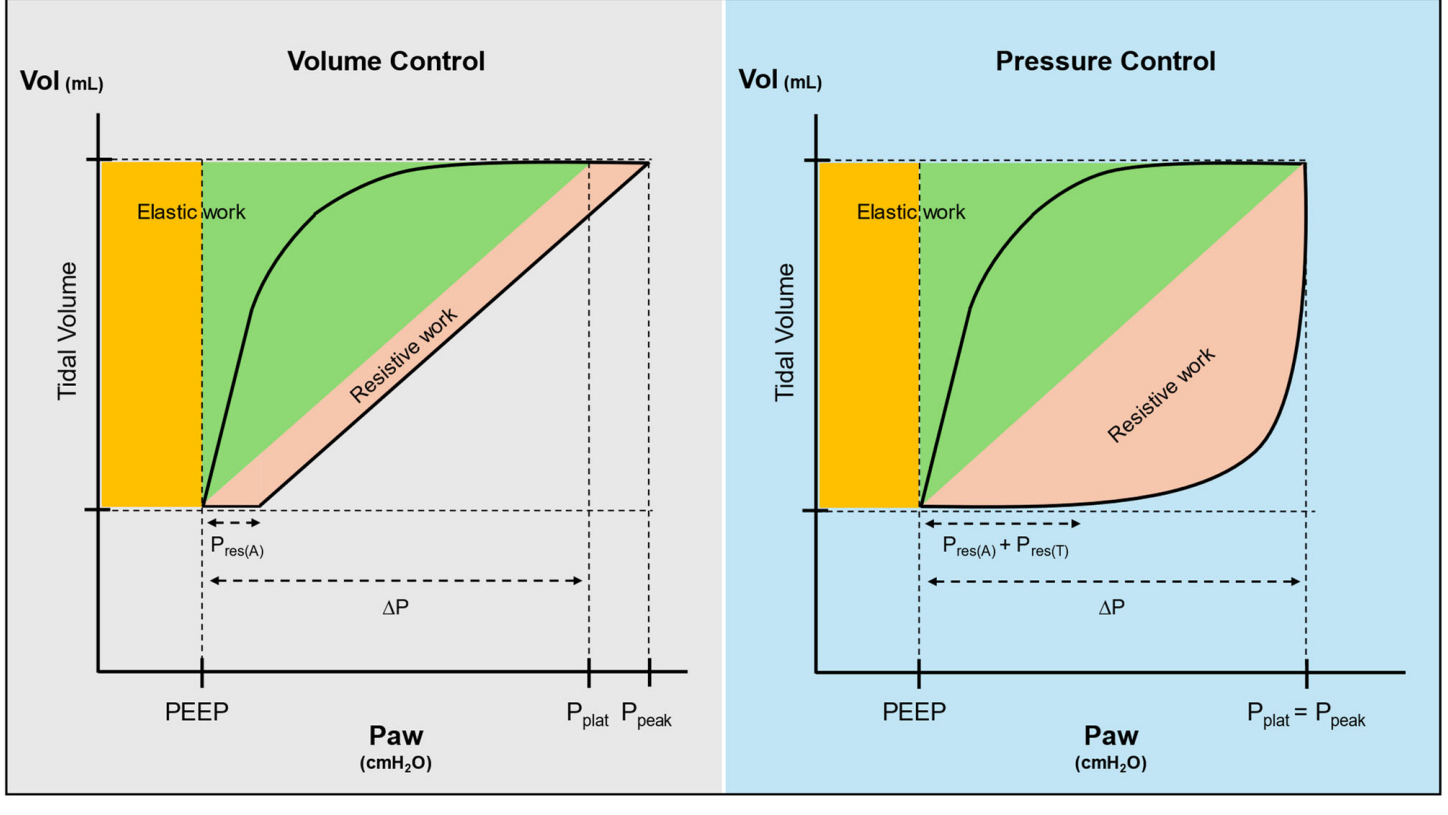
Mechanical power in volume and pressure control ventilation. Note the different shapes of the dynamic pressure–volume curves under volume control with constant (square) flow and pressure control with decelerating flow. The greater resistive work (pink area) in pressure control ventilation is due to the higher initial flows. To highlight this phenomenon, we have assumed here that tissue resistances play a significant role only in pressure control ventilation, although they certainly (but at a lower level) exist in volume control ventilation as well. The green and yellow areas represent the elastic work due to PEEP and driving pressure and are not different in the two modes of ventilation. The sum of the yellow, green, and pink areas describes the inspiratory work, from which the mechanical power is currently calculated. Conversely, the area (pink + green) enclosed by the inspiratory and expiratory limbs (solid black lines) represents the hysteresis area, indicating the dissipated energy that remains in the parenchyma after a whole breath. Please note that the pressure–volume curve describing mechanical power is *dynamic* (i.e., at clinical inspiratory flow), as opposed to the *low-flow* pressure–volume curve described in Figure 2 to assess recruitability. Paw: airway pressure, Vol: volume, P_res(A)_: airway resistive pressure, ∆P: driving pressure, PEEP: positive end-expiratory pressure, P_plat_: plateau pressure, P_peak_: peak inspiratory pressure, P_res(T)_: tissue resistive pressure.

When assessing the inspiratory and expiratory phases in isolation, the impact of mechanical ventilation on the entire breath is not appreciated. Moreover, the relevance of respiratory rate (RR) in terms of VILI remains underappreciated. For this reason, the concept of mechanical power (MP) has received great attention [46]. The idea is that what causes lung damage is the total energy transferred to the parenchyma by the ventilator and how often this energy is delivered (power = energy per unit of time). The energy-per-breath is represented by the area under the inspiratory limb of a *dynamic* PV loop (Figure 3) and can be calculated at the bedside using equations that vary depending on the mode of ventilation employed [47,48,49]. For square wave volume-controlled ventilation, the MP can be estimated as:

(5) MP=0.098·RR·Vt·(Peak inspiratory pressure−1/2(ΔP))

For pressure-controlled modes, the MP can be estimated as:

(6) MP=0.098·RR·Vt·Peak inspiratory pressure

The concept of MP has added significant knowledge to our understanding of VILI. Most importantly, it is now accepted that relatively low ∆P or Vt can be associated with severe lung injury if delivered at high RR [50,51]. Moreover, for the same ∆P, Vt, and RR, MP increases with inspiratory flow, a previously neglected variable in the assessment of VILI risk. Flow *per se* is associated with lung injury, likely due to the time-dependence of viscoelastic tissue resistance and associated dissipation of energy inside the lung parenchyma [52]. This could raise concerns in the use of high, decelerating flow modes of ventilation (pressure control) [53,54], compared to lower, constant flow modes (volume control), as MP might be higher at similar PEEP, ∆P, and Vt [54]. Finally, MP underlines the potential risks of PEEP, as under experimental conditions lung injury worsens with increasing PEEP when all other ventilatory variables remain unchanged [55].

Despite these merits, MP suffers from some limitations: *first*, except for retrospectively evaluated thresholds possibly predicting outcome [2], a clear pathophysiological threshold for damage is not yet clear [3,56]. It can be hypothesised, however, that energy insults only exceed the parenchyma’s capacity for repair beyond a certain threshold, and that VILI develops depending on the duration spent above this level [57]; *second*, MP takes into account airway resistive components, which might be questionable in terms of VILI [58]; *third*, it remains unclear whether the actual damaging energy is described by the area under the inspiratory limb of the PV loop or by the hysteresis area enclosed between the inspiratory and expiratory limbs: the latter represents the dissipated energy that remains in the parenchyma after a breath, and it may be more closely related to VILI than the total energy input [59,60]; *finally*, energy, like volume, is an *extensive* variable. Accordingly, MP should be scaled to the baby lung size to obtain an *intensive* parameter applicable in different patients. MP normalised to C_stat_ has indeed been used in retrospective analyses, with better performance than standard MP in predicting outcome [61,62].

### 2.4. Understanding the Contribution of the Chest Wall

All the ventilatory variables described above, which are available *at a glance* from a ventilator, refer to the respiratory system as a whole, i.e., lung and chest wall. However, only the former is relevant to VILI, therefore, partitioning respiratory mechanics into lung and chest wall components should be a skill in the hand of any physician dealing with mechanically ventilated patients [16].

In the previous sections, we defined strain (Equation (2)) as the change in lung volume above its resting volume (FRC + V_rec_) [33]. We also discussed that a clinical proxy measure of the resting lung volume is represented by C_stat_, which helps convert ventilatory variables from extensive (e.g., Vt) into intensive (e.g., ∆P) by “normalising” them to lung size. However, C_stat_ refers to the whole respiratory system, i.e., lung and chest wall in series. Since the respiratory system elastance (i.e., the opposite of compliance) is the sum of lung and chest wall elastances (E_RS_ = E_L_ + E_cw_), a low C_stat_ (i.e., high E_RS_) might indicate a high E_CW_ rather than E_L_, therefore not reflecting a small resting lung volume. In this case, intensive ventilatory variables derived by C_stat_ (e.g., ∆P, PEEP) may not accurately reflect lung strain, which is the real determinant of VILI [63,64].

Evaluating lung strain is challenging in clinical practice. Accurate measurement of resting lung volumes (FRC + V_rec_) to calculate Equation (2) requires offline analysis of CT scans, electrical impedance tomography, or use of rarely available techniques like helium dilution and nitrogen wash-out–wash-in [65]. Therefore, one must rely on the relationship between strain and stress, the latter representing the reactive force per unit of area that develops within a material in response to the application of an external force [16]:

(7) Stress=K·Strain

In the lung, stress is the transpulmonary pressure, i.e., the proportion of airway pressure that distributes to the lung and not to the chest wall, while the constant K is the specific elastance (E_S_), reflecting the intrinsic elasticity of alveoli and defined as the transpulmonary pressure required to double the FRC. While differences exist between species, E_S_ is fairly constant in healthy and diseased human lungs (~12–13 cmH_2_O), therefore strain can be derived if transpulmonary pressure is known. For instance, a transpulmonary pressure equal to 25 cmH_2_O at the end of inspiration corresponds to a strain ~2, a value that approximately reflects total lung capacity [33]. Calculation of transpulmonary pressure is challenging, as it requires oesophageal manometry to estimate the pleural pressure [16] (Figure 1). It is important to emphasise that *absolute values* of oesophageal pressures are influenced by oesophageal catheter or body position and body weight, therefore its dynamic *changes* might be more reflective of changes in pleural pressure [31,66]. However, this remains debated, and two methods exist to calculate transpulmonary pressures: the first one (referred to as the “direct method”) subtracts *absolute* values of oesophageal pressures from airway pressures, while the second one (the “elastance-derived method”) uses tidal *changes* in oesophageal pressure to calculate the ratio of lung to respiratory system elastance (E_R_ = E_L_/E_RS_) and converts airway pressures (namely, P_plat_, ∆P, PEEP) into transpulmonary pressures (respectively, P_L_, ∆P_L_, PEEP_L_) by simply multiplying by E_R_ [67] (Figure 1). The first method is generally used to set PEEP to a value that allows for a slightly positive (0–2 cmH_2_O) end-expiratory transpulmonary pressure (i.e., PEEP *minus* end-expiratory oesophageal pressure), therefore avoiding collapse of dependent lung regions. The second method is more relevant in defining safety thresholds for inspiratory pressures and inspiratory stress [67] (e.g., P_L_ < 20–22 cmH_2_O [68] or ∆P_L_ < 10–12 cmH_2_O [69]), avoiding overdistension of non-dependent lung zones. For the clinician, it is important to keep in mind that (1) stress (i.e., the *transpulmonary* pressure) rather than *airway* pressure relates to lung damage, as it is proportional to strain; (2) oesophageal pressure, despite its limitations, is the best clinical surrogate of pleural pressure; (3) for the same E_RS_, the distribution of airway pressure to the lung decreases as E_CW_ increases; (4) an end-inspiratory strain ~2 reflects inflation to almost total lung capacity and should be considered harmful; (5) a positive end-expiratory transpulmonary pressure might be targeted to avoid lung collapse.

## 3. Spontaneously Breathing Patients Under Assisted Ventilation

Now we shall examine the assessment of patients making respiratory efforts. In patients awake or lightly sedated and ventilated in assisted modes the interaction between the ventilator and the lung parenchyma is complicated by the patient’s own respiratory effort. The latter reflects how the neural respiratory drive (i.e., the output from the respiratory centres) translates into contraction of the diaphragm and other inspiratory muscles. Accordingly, a proper assessment of the respiratory system must consider indices of drive, effort, and muscular activity during assisted modes. In the remainder of the article, we will focus on pressure-controlled modes with a fixed level of assistance (e.g., a set “pressure support”). Indeed, these are the most commonly used modes in the ICU where patients are transitioning towards spontaneous breathing or extubation [70,71].

### 3.1. Assessing Neural Respiratory Drive

**Figure 4:**
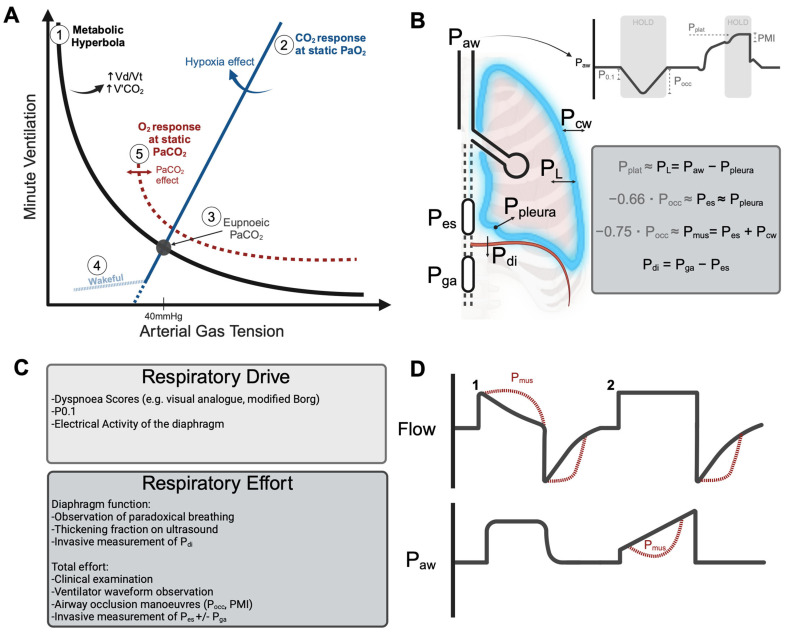
Respiratory Drive and Effort. Panel (**A**): Control of breathing. 1: The metabolic hyperbola describes changes in minute ventilation and PaCO_2_ depending on its total metabolic production (V’CO_2_) and the dead space (Vd/Vt). 2: The ventilatory response to PaCO_2_ (in L/min/mmHg of PaCO_2_) relative to the eupnoeic PaCO_2_. This slope is affected by hypoxia, distress, inflammation. 3: The eupnoeic PaCO_2_ is the intercept between the CO_2_ response curve and the metabolic hyperbola. It may be shifted by hypoxia. 4: In a wakeful state apnoea does not normally occur whilst during sleep or sedation apnoea occurs when the actual PaCO_2_ < eupnoeic CO_2_. 5: The ventilatory response to PaO_2_ is minimal, >60 mmHg (with normal PaCO_2_), below which it exponentiates. This curve resembles an inverted oxygen–haemoglobin dissociation curve. Panel (**B**): Pressures related to respiration and their relations. A positive transpulmonary pressure (P_L_) is required to generate inspiratory flow. This can occur through P_mus_ (leading to ↓ P_pleura_) or through Pvent (↑ P_aw_) or a combination. Pressures measured during inspiration against a closed valve (P0.1, P_occ_) or during an end-inspiratory hold (P_plat_, PMI) can be used to estimate invasive measurements. Panel (**C**): Assessments of respiratory drive and effort. Panel (**D**): Breath 1 is pressure controlled: P_mus_ is easiest to see on the flow waveform where it exceeds the exponential decay seen during a passive inspiration or expiration (black line). Breath 2 is volume controlled, whereby inspiratory P_mus_ is more obvious on the pressure scalar.

The brainstem centres generate an oscillatory output from continuous tonic inputs from peripheral chemoreceptors (sensing pH, PaO_2_, oxygen delivery, and PaCO_2_), central chemoceptors (sensing pH), airway, lung, chest wall, and peripheral mechanoceptors, bronchopulmonary c-fibres, trigeminal nerve sensory fibres, and descending inputs from the cerebral cortex (which normally exert an inhibitory effect) [72,73]. The relationships between the inputs to the respiratory centres and the resulting respiratory drive during critical illness have been reviewed in detail elsewhere [73]. To summarise, the normal physiological response is to tightly control the PaCO_2_ at a set point determined by the respiratory centres—the “eupnoeic PaCO_2_” (Figure 4). The eupnoeic PaCO_2_ is influenced by the presence of inflammation, hypoxia, pain, distress, and mechanoceptor feedback regarding the elastance of the respiratory system. This accounts for the common occurrence of hypocapnia—a reduction in the eupnoeic set point—in patients with acute hypoxaemic respiratory failure. Importantly, a given PaCO_2_ set point will be maintained unless the ventilation demanded by the respiratory centres exceeds the patient’s capacity to achieve it (e.g., due to the presence of relevant dead space or muscle weakness), with or without mechanical assistance. For instance, during exercise, despite vast increases in the metabolic production of CO_2_, there is minimal change in PaCO_2_ due to an enormous increase in respiratory drive to maintain the eupnoeic set point [74].

#### 3.1.1. Clinical Assessment of Neural Respiratory Drive

1. *Blood gases*

Given the above, as the spontaneously breathing patient will maintain their eupnoeic PaCO_2_ with changes in effort over time, it follows that normocapnia is not a reliable guide to the respiratory drive [73]. Accordingly, a PaCO_2_ of 45 mmHg may reflect normal respiratory drive, blunted respiratory drive (e.g., under sedation), or highly elevated respiratory drive with incipient exhaustion (e.g., life-threatening asthma).

2. *Respiratory* *rate*

Although widely used, trending the respiratory rate also has limitations. Significant tachypnoea usually occurs when drive is increased 3–4 times above normal and the respiratory rate (as opposed to minute ventilation) is stable over a wide range of PaCO_2_ and changes in metabolic CO_2_ production [73,74,75,76]. This is because the predominant effect of increased respiratory drive is an increased output-per-breath rather than an increased frequency of output (i.e., respiratory rate) [76].

3. *Dyspnoea*

Dyspnoea is one of the most distressing human experiences and is common during mechanical ventilation, present in up to 50% of patients [72]. Although the pathophysiology of dyspnoea is not yet fully understood, it is postulated to result from a mismatch between neural respiratory drive and ventilation, i.e., between the output from the respiratory centres and the actual minute volume that the respiratory system can achieve [72,73]. Accordingly, patients with high respiratory drive may not perceive any dyspnoea if the respiratory system is intact (e.g., during compensatory hyperventilation in metabolic acidosis, Kussmaul breathing [77]), while reporting high discomfort when lung compliance is decreased (e.g., in pulmonary oedema). It must be noted, however, that dyspnoea is a complex emotional state which might also be influenced by abnormal cortical interpretation of normal sensory inputs (e.g., in hyperventilation syndrome) or be masked by sedation even with very abnormal respiratory centre inputs (e.g., in the ICU). In any case, it must derive partly from afferent cortical inputs from the brainstem respiratory centres.

Recent consensus statements have emphasised proactively asking intubated patients about dyspnoea (“does your breathing feel comfortable?”) [72]. Several clinical scores to evaluate for dyspnoea have been used, including the (modified) Borg scale and the use of visual analogue scales. Interestingly, around 40% of patients who deny dyspnoea score their current level of dyspnoea as >0 when prompted [78]. Of course, such assessments may be challenging in the presence of sedation and delirium. Observation scales have been proposed to assess for dyspnoea in patients who are not able to reliably communicate [72], but in practice these rely on clinical signs of high effort—thereby they may be insensitive (see below).

#### 3.1.2. Invasive Assessment of Neural Respiratory Drive

The electrical activity of the diaphragm (EA_di_) can be measured to provide a real-time breath-to-breath assessment of neural respiratory drive [79]. Measuring EA_di_ requires insertion of a dedicated nasogastric catheter (or less often, surface or needle electromyography) from which a filtered signal can be derived [80]. Although activity <10 mV generally indicates abnormally low respiratory drive, there is significant variation in high values between individuals [80], which limits designation of strict thresholds or direct correlations with muscular activity. However, changes over time within individuals are a reliable assessment of drive. Electromyography of other respiratory muscles has also been described [81]—though stimulation of accessory muscles can only indicate high drive.

#### 3.1.3. Ventilator Assessment of Neural Respiratory Drive

The pressure with a transient valve closure at 100 ms following the onset of inspiration (P0.1) (Figure 4) is a widely used surrogate metric for respiratory drive. This transient valve closure is designed to occur quickly enough that the respiratory centres do not react to it. Targeting a range of −1.5 to −3.5 cmH_2_O has been suggested [82,83], with values less than around −5 to −10 cmH_2_O suggesting extremely high respiratory drive. Recently, P0.1 < −3.5 cmH_2_O was found to be associated with patient-reported dyspnoea, increased duration of ventilation, and even mortality [84]. The strength of this metric is that it can be automated on almost all ICU ventilators and can be trended over time. It has been demonstrated that P0.1 remains reliable even when *partial* neuromuscular blockade is progressively added to induce significant weakness [85] although it is conceivable that profound muscle weakness could affect it in some patients.

### 3.2. Assessing the Diaphragm

The diaphragm is the primary muscular pump to generate flow into the respiratory system, especially for mechanically ventilated patients who are generally supine. Consequently, there is a more established literature regarding its assessment than for the other respiratory muscles. Separating the thoracic and abdominal cavities, diaphragm muscle fibre shortening causes diaphragm thickening and descent, expanding the thorax and decreasing pleural pressure to generate inspiratory flow [82,86,87]. If the diaphragm is healthy, recruitment of accessory muscles occurs only with extreme elevations in respiratory drive and effort [81]. Conversely, diaphragm dysfunction (most extreme in cervical cord injury) necessitates recruitment of accessory muscles for tidal ventilation.

Diaphragm dysfunction is common in the critically ill [86,87,88]. Although certain disease-related risk factors are shared with “critical-illness-acquired weakness”, in fact there is poor correlation between the two in individuals. Up to 80% of patients who receive prolonged ventilation have been found to have a degree of diaphragm dysfunction [86]. This may be because mechanical ventilation predisposes the diaphragm to injury [82,87,88]. In passively ventilated patients, histological atrophy from disuse occurs within hours to days [89]. Conversely, if the demands on the diaphragm are persistently excessive, “exertion myotrauma” may result in sarcomere disruption, inflammation, and injury [86,87]. Asynchrony during mechanical ventilation can cause eccentric loading of the diaphragm leading to impairment [86,90]. Finally, excessive PEEP mechanically disadvantages the diaphragm and may lead to longitudinal atrophy [91].

#### 3.2.1. Clinical Assessment of Diaphragm Function

Clinically, diaphragm dysfunction can be suspected when there is disproportionate accessory muscle use at rest, shallow tachypnoea, or a paradoxical breathing pattern [86,92]. In the latter, abdominal wall retraction is seen during *inspiration* due to elevation of the anterior chest wall by accessory muscle activation. Orthopnoea may be a feature due to compression of the thorax by the abdominal contents in the supine position. Although hemidiaphragm palsy can be suspected on chest X-ray [92], critical-illness-associated diaphragm dysfunction is usually a bilateral process.

#### 3.2.2. Imaging Assessment of Diaphragm Function

The diaphragm can be assessed by evaluating its ability to contract on imaging. Although diaphragm descent can be assessed using dynamic fluoroscopy [92], the main imaging technique is point-of-care ultrasound. Most commonly the relative thickening fraction during inspiration vs. expiration is measured [87]. A thickening fraction of 15–30% is similar to normal breathing at rest and obtaining this degree of activity during assisted ventilation has been associated with shorter durations of ventilation [88]. The distance the diaphragm descends during inspiration can also be measured, but this is unreliable with positive pressure ventilation (which may cause passive descent) [87]. Key limitations of US are the need for operator expertise and that diagnostic views are more often obtained on the right due to the better ultrasound window afforded by the liver.

#### 3.2.3. Invasive Assessment of Diaphragm Function

The pressure generated by diaphragmatic contraction (P_di_) can be directly measured as the difference between the pressure in the stomach (P_ga_) and oesophagus (P_es_) during inspiration [86,87,92]. However, this requires insertion of a dedicated catheter with two balloon manometers. Nevertheless, this is commonly used in research studies to objectively diagnose diaphragm weakness (<70–80 cmH_2_O with maximal effort) [87]. Neuromechanical uncoupling, i.e., a dissociation between neural output and the capacity of the diaphragm to produce the required force to achieve the desired ventilation (as may happen with diaphragm flattening at high PEEP), can be assessed by the ratio of EA_di_ (see above) to P_di_ [87]. Although specific measurement of P_di_ is appealing, in clinical practice it is more common to rely on the pressure exerted by all the respiratory muscles in concert (P_mus_, see below) to assess global respiratory effort. More comprehensive parameters include the tension time index ($TTI=\frac{P_{di}}{P_{di(maximal\;effort)}}\;\cdot \frac{Inspiratory\;Time}{Duty\;Cycle\;Time}$), which takes into account the effects of muscle weakness and respiratory rate/pattern, but clinical use is limited by the challenges of obtaining maximal efforts in sedated patients [86,87].

### 3.3. Assessing Total Respiratory Effort

Respiratory effort represents the translation of neural drive into inspiratory muscular contraction, which depends on the integrity of the descending pathway from the brainstem to the respiratory muscles. It can be defined as the overall force generated by all the respiratory muscles during inspiration or muscular pressure (P_mus_, see below).

#### 3.3.1. Clinical Assessment of Respiratory Effort

Just as respiratory rate is insensitive to high respiratory drive, it is also not particularly useful as a means of judging the effort-per-breath. Whilst respiratory rate and pattern (inspiratory time, ratio of frequency to volume) are correlated with inspiratory effort during assisted ventilation, they are poorly predictive [93]. Furthermore, the respiratory rate is insensitive to excessive ventilatory support—even when the effort-per-breath is minimal [76]: although, under sedation or during sleep, apnoea can occur if mandatory ventilation is sufficient to lower the PaCO_2_ below the “apnoea threshold” [73], this occurs abruptly following breaths at a constant rate but minimal P_mus_ rather than after progressive bradypnea [76]. Overall, although a high respiratory rate requires assessment, moderate rates are not a reliable guide that P_mus_ is appropriate—it may be low, normal, or excessive (e.g., in a patient receiving an opiate infusion with high tidal volumes at a low rate).

Clinical examination can identify signs suggesting high breathing efforts such as diaphoresis, nasal flaring, phasic sternocleidomastoid activity, tracheal tugging, suprasternal notch recessions, intercostal retractions, and abdominal muscle activation [81,94,95]. All of these may be observed in patients during mechanical ventilation when efforts are high. However, interobserver correlations are only moderate [96], many signs are only apparent at severe levels of effort [97], and accessory muscle use has been more often assessed with needle or surface electromyography than palpation in studies [98]. Accordingly, clinical examination may be best suited as a screening tool to identify only extreme levels of efforts.

#### 3.3.2. Invasive Assessment of Respiratory Effort

If an oesophageal balloon catheter is placed, an intrathoracic oesophageal pressure can be measured (P_es_) which closely approximates basal pleural pressure (P_pleura_) (Figure 4) [99]. This waveform will display negative pressure swings during spontaneous inspiratory efforts referred to as the ΔP_es_ [100]. This is well correlated to the pressure exerted by the respiratory muscles, P_mus_ (ΔP_es_ plus the pressure required to overcome the elastic recoil of the chest wall).

During normal breathing at rest, a ΔP_es_ value of around 3 cmH_2_O can be anticipated [99]. During mechanical ventilation, a ΔP_es_ < 5 cmH_2_O and certainly <3 cmH_2_O is considered to raise concern for “overassistance” and the risk of respiratory muscle atrophy [82]. In contrast a ΔP_es_ > 15 cmH_2_O suggests very high efforts [88,101,102]. To put this in a more relatable context, a ΔP_es_ of around 16 cmH_2_O has been obtained during cardiopulmonary exercise testing at the anaerobic threshold [103]. High ΔP_es_ as ventilatory support is changed correlates with subsequent clinical failure across a wide range of illness severities, support strategies, and ages [104,105,106,107,108,109]. Notably, unrecognised strenuous inspiratory efforts lead to patient self-inflicted lung injury (P-SILI) [110,111] even when the global lung strain appears safe (see below). This is due to the pendelluft phenomenon [112,113], which creates areas of increased regional lung strain in the presence of severely inhomogeneous lung.

#### 3.3.3. Ventilatory Assessment of Respiratory Effort

The ventilator offers some possibilities to non-invasively assess respiratory efforts using surrogates of ΔP_es_:

1. *Occlusion pressure*

After complete expiration, if the valve is held closed for an entire inspiratory cycle, the decrease in airway pressure reflects the change in P_es_ (Figure 4) (i.e., similar to the measurement of plateau pressure, absence of flow means the change in airway pressure equilibrates with the change in pleural pressure). The maximal inspiratory tidal change from PEEP during a hold manoeuvre is referred to as the occlusion pressure (P_occ_)—this necessarily has a negative value (or alternatively can be referred to as a positive value (ΔP_occ_)) [101].

Several empirical studies have now been performed which have validated this measure in adults and children, independently validating the constant’s ability to infer the total effort (ΔP_es_ = −0.66 · P_occ_; or P_mus_= −0.75 · P_occ_) [101,102,114]. P_occ_ is easy to perform and is very reliable to estimate when efforts are very high (ΔP_occ_ of at least 20 cmH_2_O equates to P_mus_ > 15 cmH_2_O) or very low (ΔP_occ_ of less than 7 cmH_2_O equates to P_mus_ < 5 cmH_2_O) [102]. In a paediatric population, P_occ_ tends to be less influenced by patient age than P0.1 [114].

2. *Negative inspiratory force*

Asking the patient to make maximal effort against a closed valve is useful to measure the negative inspiratory force (NIF) [115] (i.e., the maximal attainable ΔP_occ_ rather than a breath-to-breath value). Although this is not an index of the respiratory effort that patients are experiencing at every breath, where significant respiratory muscle weakness is suspected, indexing ΔP_occ_ to this value helps to understand the burden of effort per breath (e.g., breath-to-breath effort 40% or 90% of maximal). Conversely, peak expiratory flow during a forced expiration (analogous to cough peak flow in non-intubated patients) can be measured. While primarily used for weaning assessment (values < 60 L/min associated with greater reintubation risk), reduced values may also indicate expiratory muscle weakness or increased airway resistance [116].

3. *Pressure Muscle Index*

During an end-inspiratory hold with spontaneous effort, the plateau pressure may be *higher* than the peak pressure if there is significant inspiratory effort by the patient. This occurs as relaxation of the inspiratory muscles against a closed valve produces a positive recoil pressure which is proportional to their efforts (Figure 4) [117]. Of note, in some cases expiratory muscle activation during valve closure renders the plateau pressure unreliable, however, it is usually possible to obtain an accurate plateau pressure (where there is a clear, flat plateau) over a few attempts [118]. The difference between the plateau pressure and the peak pressure is called the pressure muscle index (PMI) with values > 6 cmH_2_O suggesting high inspiratory efforts are being made [117] (including in paediatric patients [114]). Importantly, the difference between the plateau pressure during this manoeuvre and the PEEP is a closer correlate of the transpulmonary driving pressure than the difference between peak and plateau pressures (which ignores the contribution of the patient) [1]. Overall, although there is less extensive evidence regarding the meaning of driving pressure obtained in this manner (as the elastance of the chest wall varies during spontaneous breathing) [119], end-inspiratory holds during pressure support generate plateau pressures which are highly correlated with the passive values at similar tidal volumes and flows [120]. However, the driving pressure obtained by this method is unreliable if patients are making significant *expiratory* muscle effort, as expiratory effort reduces end-expiratory transpulmonary pressure below that generated by PEEP [121] (i.e., in this setting the true driving pressure exceeds the measured one [122]). Importantly, although expiratory efforts are most prominent in patients with COPD and asthma, they are also a well-recognised phenomenon in response to PEEP in patients undergoing surgery or during ARDS and this effect can be potentiated by fentanyl [123,124].

### 3.4. Assessing the Balance Between Ventilator and Respiratory Efforts

When a breath involves pressure generated by the ventilator (P_vent_) and by all the patient’s respiratory muscles (P_mus_) over time, the equation of motion describes their relationship with changes in volume (V) (depending on the elastance of the respiratory system (E)) and flow ($\dot{V}$) (depending on the resistance to airflow of the airways and apparatus (R)):

(8) $$\Delta P_{vent}+{\Delta P}_{mus}=E\cdot \Delta V+R\cdot \dot{V}$$

Accordingly, although the ventilator displays P_vent_, $\dot{V}$, and the tidal volume, assessing P_mus_ is needed to understand whether the tidal or total stress being applied to the lung (the transpulmonary pressure, P_L_) is injurious. This is because the P_L_ may be high despite an acceptable P_vent_ if there is sufficient P_mus_ (Figure 4) [16]. Similar to passive modes, the P_L_ can be calculated from invasive measurements of P_es_ using the elastance-derived method (Figure 1), and maintaining a total end-inspiratory P_L_ of <20–22 cmH_2_O and ΔP_L_ of <10–12 cmH_2_O are targets to prevent VILI [16,99,101,102]. Alternatively, an estimated transpulmonary pressure can be calculated using P_occ_ (EstP_L_ = P_peak_ + (−0.66 · P_occ_)) [101,102,114], a method which can accurately identify when the P_L_ is >20 cmH_2_O (EstP_L_ > 22 cmH_2_O).

**Figure 5:**
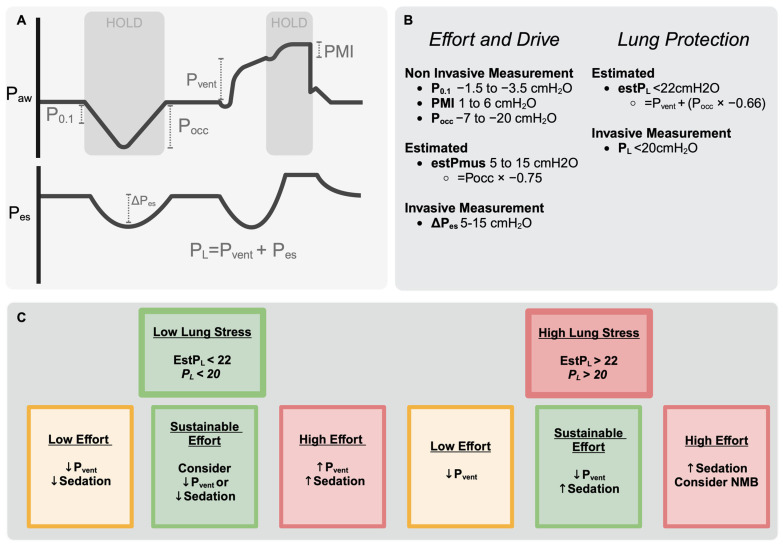
Aims for lung and diaphragm protective ventilation. (**A**): Either non-invasive intermittent techniques (above) or continuous invasive monitoring of P_es_ and P_L_ can be used (below). Non-invasive techniques during inspiration either against a closed valve (P0.1 and Pocc) or at end inspiration (PMI). When valves are closed and there is no flow, pressures measured at the airway approximate pleural pressures during active efforts (P0.1, Pocc). Alternatively, an end-inspiratory hold allows the relaxation pressure of the inspiratory muscles to be measured (PMI). (**B**): Either invasively measured or estimated measures of respiratory effort and transpulmonary pressure can be used. Relevant calculations together with suggested targets are listed. (**C**): Selected strategies to achieve a sustainable degree of respiratory effort alongside a protective level of lung stress are shown, depending on whether the total stress applied to the lung is high or acceptable and on the degree of respiratory effort.

Figure 5 displays an approach to titrating ventilatory assistance and sedation depending on the estimates of P_mus_ and P_L_ [102]. To understand this approach, one should remember that if patients make consistent spontaneous effort, and oxygenation and sedation are held static, changes in pressure support do not generally lead to changes in PaCO_2_ or minute ventilation [73,120]: if ventilatory assistance is reduced, spontaneously breathing patients will increase their inspiratory efforts to maintain their PaCO_2_ set point, therefore the total transpulmonary pressure (P_L_) remains static [120,125]. It should also be noted that although increasing sedation is advocated in cases of increased lung stress (Figure 5), the correlation between depth of sedation and respiratory drive is relatively weak in acutely ill patients [126] and occasionally a return to mandatory ventilation is merited for lung protection.

## 4. Conclusions

Mechanical ventilation is an effective supportive therapy in both the operating theatre and the ICU. However, it carries a significant risk of lung and diaphragm injury which must be considered and minimised. Learning to properly assess the respiratory system under conditions of both mandatory and assisted ventilation is necessary to this end. We encourage management guided by *intensive* variables like ∆P, ideally scaled to the isolated lung through oesophageal manometry when abnormal chest wall elastance is suspected. We highlight the need for a physiologically based assessment of the effects of PEEP on recruitment and overdistension. Until more data are available on safety thresholds for MP, we focus on the relevance of respiratory rate and inspiratory flow as contributing factors to lung damage. Finally, we stress the relevance of ensuring adequate respiratory effort to minimise diaphragm dysfunction and dyspnoea.

## Figures and Tables

**Table 1:** Extensive vs. intensive ventilatory variables.

Extensive	Intensive
Tidal Volume ($V_{t}$)	Driving Pressure (∆P = $V_{t}$/$C_{stat}$)
PEEP-induced inflation volume (V_infl_)	PEEP (PEEP = V_infl_/$C_{stat}$)
PEEP-induced recruited volume (V_rec_)	PEEP (PEEP = V_rec_/C_rec_)
Compliance ($C_{stat}$)	Specific compliance (C_s_ = $C_{stat}$/FRC)
Elastance (E)	Specific elastance (E_s_ = E ∙ FRC = Stress/Strain *)
Mechanical power (MP)	Normalised MP MP_norm_ = MP/$C_{stat}$

Note that intensive variables result from the ratio of extensive or intensive variables. C_rec_: compliance of the recruited volume, see text, FRC: functional residual capacity. * Stress and strain are both intensive variables.

## Data Availability

Not applicable. No dataset was used for this review.
